# Improving wasting among children under-5 years in Malawi: The role of farm input subsidies

**DOI:** 10.3389/fpubh.2022.862461

**Published:** 2022-09-07

**Authors:** Grace Tione, Edith Gondwe, Beston B. Maonga, Kennedy Machira, Samson Pilanazo Katengeza

**Affiliations:** Department of Agricultural and Applied Economics, Lilongwe University of Agriculture and Natural Resources, Lilongwe, Malawi

**Keywords:** FISP, wasting, child malnutrition, dietary diversity, correlated random effects

## Abstract

Wasting among children under-5 years remains a public health problem in Malawi, despite the quest to improve food availability through Farm Input Subsidy Program (FISP). As such, the study examined the link between FISP and child wasting. Using Malawi Integrated Household Panel Surveys for 2013, 2016, and 2019, two-stage least squares approach was employed to run a Cobb Douglas production function and a correlated Random Effects (CRE) Model to account for endogeneity challenges and an unbalanced panel dataset. The study hypothesized the role of FISP to dietary diversity at the household level on child wasting [weight-for-height (WHZ)]. Based on the analysis, the study found that household access to FISP coupons was not a stand-alone predictor for low wasting among children under-5 years. However, increased maize production due to FISP coupon access significantly correlated with lower wasting likelihood incidences at the household level. Worth to note, that in 2015/16, households that had accessed FISP coupons and were in the central region had higher wasting probabilities among the children under-5 years in Malawi compared to other counterparts panels. This implies challenges to addressing wasting among children under-5 years– which can be attributed to higher redemption costs of the FISP coupon. Therefore, the current study suggests that input subsidies can improve the reduction of wasting among children under-5 years through specific pathways, among them, increased maize production and considering appropriate targeted approaches to ensure households access the inputs for sustained food availability, which in turn enhances improved the children under-5 years health dividends in Malawi.

## Introduction

Globally, child malnutrition remains a fundamental public health challenge and is associated with undesirable health outcomes such as reduced cognitive and physical development, increased rates of sickness and death from common illnesses, and reduced educational outcomes and lifelong productive capacity (1, 2). Wasting, stunting, and underweight are expressions of under-nutrition and are anthropometric indicators for assessing a child's nutritional status (3). Recent global undernutrition estimates suggest that 149.2 million children under the age of 5 were stunted, 45.4 million wasted, and 38.9 million overweight in 2020 (2). Incidents of child undernutrition are particularly higher in Sub-Saharan Africa (SSA), which has 37 and 25% of the world's stunted and wasted children, respectively (2). The FISP has been widely critiqued, with scholars indicating that political considerations and corruption impede clear targeting of households that could make productive use of fertilizer but cannot afford to pay for it (4–6).

While causes of child malnutrition vary across geographical spaces, key determinants of malnutrition are most commonly; household food insecurity, inadequate dietary intake, diseases, low household income, lack of access to adequate clean water, insufficient health facilities, low educational level, distance to health facilities, poor hygiene, and sanitation (7–9). In addition, the incidences of child malnutrition have been further exacerbated by the global social and economic crisis caused by the COVID-19 pandemic (10, 11). The pandemic, through its negative impacts of reduced household incomes; disruption of production, transportation, and sale of nutritious, fresh, and affordable foods; and interruptions to health, nutrition, and social protection services, pose a risk to the nutritional status of children (12, 13).

In Sub-Saharan Africa (SSA), investments in nutrition policies, programs, and related advocacies to reduce child malnutrition offer important pathways to reduce negative outcomes of malnutrition (14). For example, in the case of Malawi, several programs such as exclusive breastfeeding among infants in the first 1,000 days of life have been implemented, with reported positive outcomes in reducing child nutrition-related problems (15–18). Complementary programs in the agriculture sector to reduce food insecurity and improve food diversity have also been implemented through various forms of agricultural subsidy programs (19–22). Evidence suggests that these programs can play a role in reducing child malnutrition outcomes and, therefore, the need for adequate research that informs policies.

In their various forms and contexts, agricultural input subsidies have been widely implemented in developing countries and are hypothesized to reduce malnutrition through their impacts on dietary diversity (6, 23–25). While the objectives of AIS vary widely in the Sub-Saharan region, the underlying concepts focus on higher agricultural productivity, improved food through lesser food prices, and nutrition security (6, 26).

The Farm Input Subsidy Program (FISP) in Malawi, currently known as the Affordable Input Program (AIP), is implemented to increase cereal and legumes production and provide farmers with the incentives to diversify production (21, 27–29). On a global scale, FISP also responds to the attainment of Sustainable Development Goal (SDG) 2 “End hunger, achieve food security and improved nutrition and promote sustainable agriculture” (30). The AIP program in Malawi has undergone several adjustments to align it to improved welfare outcomes. FISP was re-introduced in 2005/06 after an initial suspension of the program, consisting of fertilizer and seed inputs for maize and soybean crops (27). The core stated objective of the FISP has consistently been to improve resource-poor small-holder farmers' access to improved agricultural inputs to achieve their and national food self-sufficiency and raise these farmers' incomes through increased food and cash crop production (21).

Evidence on the performance of AIPs suggests that the overall production and welfare effects of subsidy programs tend to be smaller than expected (5, 31). Since the inception of the program, studies that have assessed the role of the FISP have focused on the contribution of FISP to coping with negative shocks (32); reduction of the gender adoption gap (33); nutrition outcomes (25); agricultural diversification (19); gendered agricultural productivity (20); and adoption of natural resource management technologies (34).

Despite the wide implementation of Agricultural Input Subsidies (AIS) and recent interest in assessing the linkages of the programs to food and nutrition security outcomes, the evidence continues to be scanty and unclear (26, 35). Like many AISs, the FISP has targeted increased productivity of agricultural households by providing input subsidies to small-scale farmers (21). In addition, the FISP program has been aligned with national development goals such as the Malawi Growth and Development Strategy (MGDS) and the global Sustainable Development Goals (SDGs) (36–38).

The prevalence of wasting among children under-5 years is generally unwanted among populations, as it reflects the short-term unavailability of food among households. While the prevalence of wasting among children in Malawi is lower than the global estimates of 6.7%, at 3.7%, the presence of wasting among children is highly undesirable. Despite the widespread use of subsidies for agricultural inputs as a crucial agricultural policy for reducing food insecurity in SSA countries, such as Malawi, discourses examining the pathways of the subsidy programs to nutrition outcomes are sparse and do not take into consideration the changing policies and implementation arrangements of the programs (35). Further, few studies link the FISP to children's outcomes (25). In this study, we address this research gap by assessing the relationship between access to FISP subsidy and the nutrition outcome of wasting among children under-5 years.

**Figure 1:**
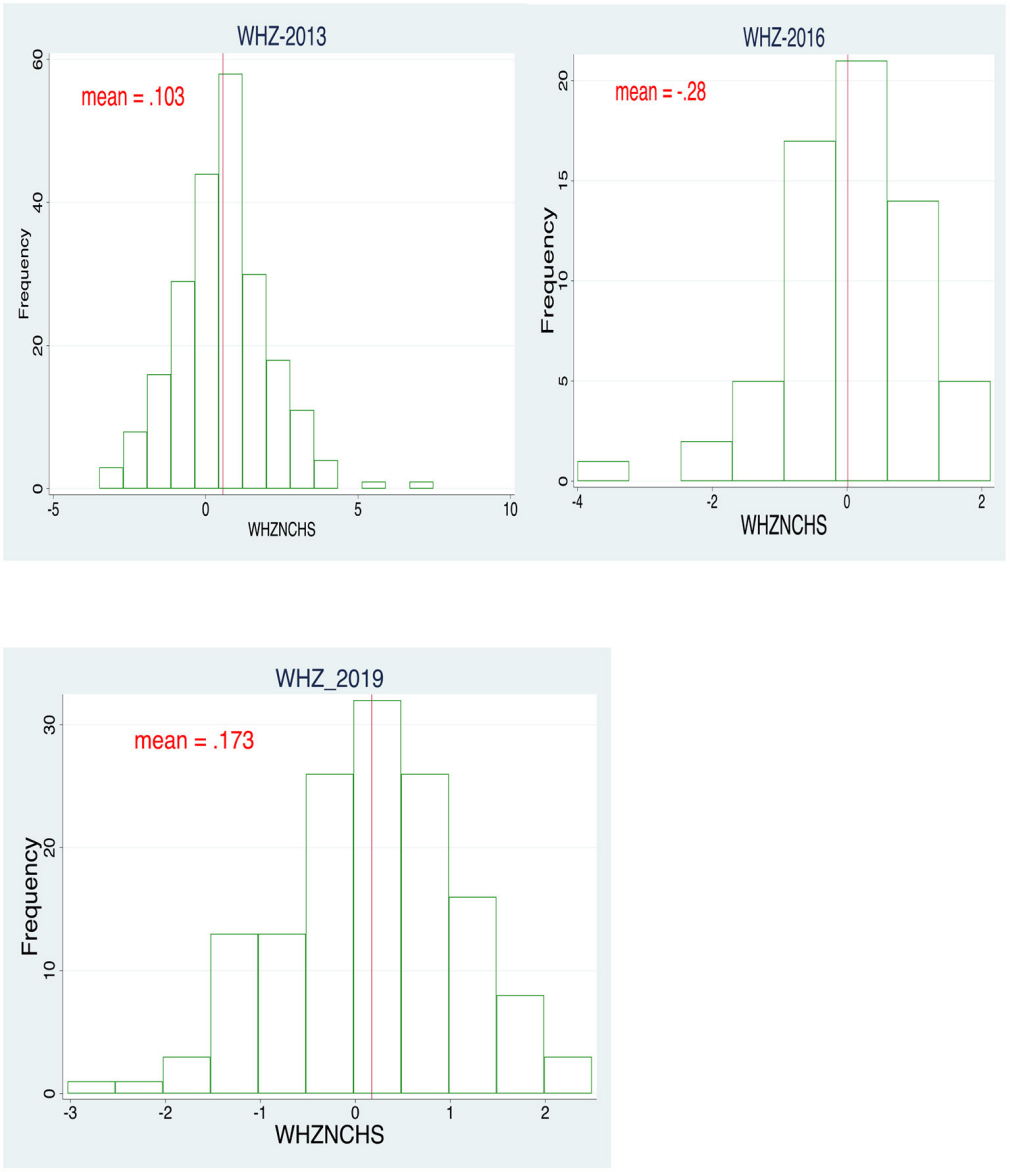
Wasting levels of children under-5 years in 2013, 2016, and 2019.

Ideally, improvements in child wasting cannot be achieved without consuming a healthy diet, and healthy food comes from either own production, gift, or purchase (39). The agricultural sector in many developing countries consists of small-holder farmers characterized by small land size (less than 3 ha) and constraints to agricultural inputs, which the Agricultural Inputs Subsidy Program (AISPs) attempt to address (5, 21). The Farm Input Subsidy Program (FISP) implemented in Malawi is this study's Agricultural input subsidy program of interest. The FISP is implemented as a social protection program that promotes food availability through subsidized inputs to increase own production and consumption of Maize and legumes (6, 19, 20). We adapt the conceptual framework offered by Walls et al. (6) and develop a conceptual framework specific for the FISP program in Malawi. Figure 1 shows wasting levels of under 5 children in the studied years.

We conceptualize FISP as being linked to child malnutrition through increased food production, especially Maize and legumes outputs, and subsequently contributing to nutritious and diversified food to children under-5 years dwelling in FISP beneficiary households. In our framework, malnutrition develops from two main immediate causes, inadequate dietary intake, and poor healthcare. In this study, we limit our analysis to the relationships between inadequate dietary intake and nutrition outcomes. Several authors have provided evidence that suggests that food insecurity, through inadequate dietary intake, is one of the major leading risk factors for child malnutrition (40–44).

FISP theoretically relates to dietary diversity through the inclusion of legumes in the program. While maize production is associated with access to starchy food sources, the inclusion of legumes is a deliberate and direct way of introducing dietary diversity in FISP beneficiary households and enhances increased food availability at the household level. The increased food access pathway is closely related to this pathway and is achieved through maize and legume production. Lastly, dietary diversity is associated with increased access to nutritious foods through household incomes. Households with disposable income realized from their crops may be better able to purchase more nutritious foods and non-food products that are imperative for dietary diversity, hence enhanced nutrition of children under-5 years.

Our study builds on the previous scholars to further establish causality between FISP and child-related nutrition outcomes. Our contribution to literature is the time dimension element whereby we used three waves of the IHP data, i.e., 2013, 2016, and 2019. It is hypothesized that previous impacts, such as those established by Harou (25), are still consistent. Our study measures the contribution of the FISP program, which has undergone significant changes in its targeting criteria (from 1.5 million farmers to 1 million farmers in 2018/2019) and increased farmers' contribution fees toward redeeming coupons, which consequently have reduced the number of beneficiaries (21). Our study is, therefore, timely in addressing these changes.

## Methods

### Study design and setting

This study used three-wave panel data from Malawi's Integrated Household Panel Survey (IHPS) for 2012/2013,2015/2016, and 2018/2019 agricultural seasons to assess the role of input subsidy in improving malnutrition in Malawi. The IHPS data is collected by the Malawi National Statistical Office (NSO) with financial support from the World Bank to monitor and evaluate changing conditions of Malawian households. The data is nationally representative and incorporates both rural and urban respondents. The surveys provide comprehensive information on households' production under agricultural modules, household consumption, income, employment, health, education, and other household characteristics under the household module and community characteristics under the community module. The selection of households is based on a stratified two-stage sample design, which firstly selects primary sampling units (PSUs) Enumeration Areas (E.A.s), and then later identifies households. A useful element to note about this dataset is that the selection criteria for beneficiaries differed in the three data collection periods. Firstly, the number of beneficiaries in 2019 was fewer than in 2013 and 2016. Secondly, the beneficiaries in 2019 and 2016 were expected to contribute MK15,000 toward the cost of fertilizer, while the beneficiaries in 2013 contributed MK4,500 (21).

#### Sampling and sample size

In our analysis, the analytical sample size of 1947 households that either received subsidy coupons in one period or did not receive input coupons. It is worthy to note that these households that received FISP coupon were selected using a targeted approach due to their poverty vulnerability attributes and identified within their community by village headmen and related community leaders (4, 45). Their community defined these households as productive poor but had land and human capital yet lacked financial capital to acquire farm inputs either timely or not.

The pooled sample size for the three periods was 1947 households. However, it is important to note that only the rural households were eligible to participate and acquire inputs under FISP. Further, not all farmers benefited from both periods (either received input coupons in 2013 or 2016, or 2019), thus creating an unbalanced data structure.

#### Children under-5 years wasting

On the malnutrition outcome, we concentrated on wasting [calculated using anthropometric measurement, Weight for Height (WHZ)]. Our choice for wasting as an anthropometric indicator was twofold. First, when severe, Wasting weakens the child's immunity system and makes the child susceptible to long-term development delays but can be reversible with urgent feeding, treatment, and care (46). Secondly, changes in wasting levels can also be observed in a short time, such as the time frame of the three-panel data periods in this study (2013-2016-2019). Children are considered moderately malnourished if they have a z-score between −3 and −2, and severely malnourished if their z-score is less than −3 (25). We further determined a child to be wasted if weight for height; (WHZ) is less than −2.

### Empirical model specification

To examine the impact of FISP in reducing child's wasting, we used two-stage least square to control for unobserved variables that cannot be controlled for but may be correlated with attributes used in selecting farming households. The first stage was to estimate Cobb Douglas production function while in the second stage, we linked the estimates in the first stage to assess whether the household that receive coupon experiences reduced wasting of its children under-5 years. It is worthy to note that our unit of analysis was at the household level.

In order to control for potential levels of endogeneity, in the first stage, we applied a Cobb Douglas production function for Maize and legume in that included production inputs in natural logs (i.e., inorganic fertilizer, organic manure, labor, land, and whether the household received input coupon in either 2012/2013 season or 2015/2016 season or 2018/2019 or one or more of them). The model was specified as follows:


(1) $$\begin{array}{l} lnM_{it}=\alpha_{0}+\gamma lnX_{it}+\theta lnZ_{it}+\mu \end{array}$$

Where *M*_*it*_ in equation 1 was output of Maize and legumes in year *t*, for household *i;* *X*_*it*_ is a vector of input variables of inorganic fertilizer, organic manure, labor, land and whether the household received input coupon in either 2012/2013 season or 2015/2016 season or both; and household characteristics were given by *Z*_*it*_. μ is the error term, while α_0_, γ* and θ* are coefficients.

In stage two, we specified the model as follows:


(2) $$\begin{array}{l} lnN_{it}\;=\;\alpha_{0}+\alpha_{1}FISP_{it}+\alpha_{2}lnM_{it}+\alpha_{3}{\left(M_{it}*FISP\right)}_{it}\\ \;+\alpha_{4}\left(year*FISP\right)+\;\alpha_{5}Z_{it}+\varepsilon_{it} \end{array}$$

### Dependent variables

The dependent variable specified as N_*it*_ is the nutrition outcome from anthropometric indicators for children under-5 years in household *i* in year *t*. The nutrition outcome of interest, wasting, measures the weight for height (WHZ) ratio of the under-five child in households *i*. We then specified children under-5 years to be wasted if z-score for variable WHZ is less than −2. A child has adequate nutrition if WHZ is between −2 and 2.

### Independent variables

The key independent variable, input subsidy (*FISP*_*it*_), was measured in two forms: whether a household is a beneficiary of input subsidies and the quantity of subsidized inorganic fertilizer used by the beneficiary household. Empirical evidence reports a positive correlation between access to subsidized inputs and the production of target crops such as Maize (47–52). Since production is an intermediary indicator of nutrition security, we expect that an increase in crop production should positively correlate with nutrition security. Thus, access to subsidized inputs is expected to positively influence nutrition security through improved agricultural production.

*M*_*it*_ controls for production of the target crop. These are maize and legume (Beans, Soya bean and Groundnuts). As discussed in the previous paragraph, we expect an increase in production of these commodities to have a significant positive influence on nutrition security. It is however not automatic that an increase in food production will result in improved nutrition security. However, naive causality cannot be assumed due to endogeneity assumptions which we discuss below. Firstly, food surpluses do not necessarily translate into access to dietary quality and nutritious food (53). Secondly, most vulnerable people in Malawi have inadequate access to calories even in years when there is a surplus of Maize (39). It is, therefore, important to establish the farm-level link of agricultural production with dietary quality and nutrition security to understand the impact of agricultural policies and programs on nutrition security (54).

We interacted maize production and quantities of subsidized inputs (fertilizer and seed). This interaction (equation 2) is represented by $M_{it}^{*}FISP$. If the coefficient of the parameter α_3_ is positive and significant, it suggests that an increase in food production due to access to subsidized inputs has a positive and significant correlation with nutrition security. Thus, access to subsidized inputs potentially enhances nutrition security through improved food crop production.

We also interacted in equation 2 above access to subsidized inputs to the year the household received input subsidy. This interaction represented as Year^*^FISP indicates the significance of receiving input subsidy in 2016 and year 2019 taking 2013 as base year. If the coefficient of parameter α_4_ is significant and negative, then wasting of children under-five years of age was improved in the households that receive input coupon in 2016 and 2019.

### Control variables

Control variables included household characteristics (Z_it_) such as age of household head, education, and household size, Where ε_*it*_ is normally distributed error term. Age of the household head assumes that the older one is, the more responsible one becomes hence likely to positively influence nutritional security. Similary, the more educated a household head, the more knowledgeable one is in terms of the child's nutritional requirements and hence enhance nutritional security by reducing Wasting among the under five.

### Data analysis and estimation

Our data analysis consisted of both descriptive and regression analysis. We analyzed key descriptive variables of access to FISP coupon, land size, maize output, legume output, household size and education. The descriptive statistics are presented as means and standard deviations. Cobb Douglas and Correlated Random Effects (CRE) regression analysis were conducted to examine the correlation of access to FISP coupons to anthropometric outcome of wasting among children under-5 years. Data was managed and analyzed using STATA 17, and all statistical analyses were performed within the program.

This CRE strategy addresses the problem of unobserved heterogeneity in the model especially with unbalanced panel data. The CRE strategy also partially address the endogeneity problem associated with access to input subsidies because the identification strategy of beneficiaries is not random. This problem is partly addressed in the CRE strategy because it controls for unobserved heterogeneity which is the main cause of the endogeneity (55). It is important to note that, we included the mean of time-invariant variables in these correlated random effects (CRE) specification.

## Results

### Background characteristics

In Table 1 through **Table 4**, we present summaries of descriptive statistics of variables used to analyze the impact of the input subsidy program in reducing wasting of children under-5 years in Malawi. Table 1 provides the distribution of the sample across the three sampling periods. We observe a decline in the number of FISP beneficiaries as we move across the three periods, with 2019 registering the lowest number of 143 from 225 in 2013. These results are consistent with the reduction in the number of beneficiaries of the FISP program (21).

**Table 1:** Study sample of the 2013_2016_2019 panels.

**Year**	**Total observations**		**Beneficiary**	**Non-beneficiary**
	**Frequency**	**%**	**N**	**N**
2013	622	31.95	225	397
2016	296	15.36	220	76
2019	1,026	52.70	143	883
Number of observations	1,947			

In Table 2, we presented the descriptive statistics of the explanatory variables. This mainly showed household characteristics, including demographic and economic variables such as land size (farm size) measured in acres, and maize and legume output measured in kilograms.

**Table 2:** Descriptive statistics of the explanatory variables.

	**2013**	**2016**	**2019**
Age	36.59	34.07	35.63
Household size	5.81	5.21	5.17
Education (years)	7.06	6.45	7.56
Maize yield (kg)	811.62	375.22	426.39
Legume (kg)	147.42	90.01	1,546.39
Land (acres)	1.79	1.56	1.53

It can be observed that the mean land size cultivated by the households decreased across the years, with a corresponding decrease in maize output (from 811.62 kg in 2013 to 426.39.30 kg in 2019). On the other hand, legume production increased from 147.42 to 1546.39 across the panels.

Table 3 presents the descriptive statistics of the explanatory variables disaggregated by beneficiaries and non-beneficiaries of FISP. We found that on average, FISP beneficiaries cultivated relatively bigger land sizes (19%) than non-beneficiaries, with a corresponding 20% higher maize output (640.58 kg) than their non-beneficiary counterparts (512.1 kg). However, non-beneficiaries produced more legumes than beneficiaries (399 % more).

**Table 3:** Descriptive statistics of the explanatory variables by beneficiary category.

**Variables**	**Beneficiary**	**Non-beneficiary**	**Pooled**
Age (Years)	37.09	35.28	35.69
Household size	5.7	5.3	5.4
Education (years)	6.9	7.3	7.2
Maize yield (kg)	640.58	512.1	541.59
Legume (kg)	215.72	1,072.52	878.04
Land (acres)	1.90	1.53	1.62

### Wasting among children under-5 years

Table 4 presents descriptive statistics of wasting among children under-5 years in Malawi. Overall, the mean Z-score is 3.66, suggesting that, on average, children under-5 years in the sampled households were not wasted.

**Table 4:** Descriptive statistics of the outcome indicator- wasting.

	**Observations**	**Mean**	**Std. Dev**.	**Min**	**MaxW**
WHZNCHS	1,947	3.668906	18.04792	−4	30

Figure 1 presents three histograms for 2013, 2016, and 2019 panels. We find fluctuations in the WHZ values across the three periods. Firstly, we observe that the WHZ value in 2013 was positively skewed (0.103). However in 2016, the wasting levels declined, with a mean WHZ value of −0.28. An improvement in the wasting levels is recorded for 2019, at 0.173.

### Impact of FISP on reducing wasting amongst children under-5 years

Table 5 presents the determinants of wasting among FISP and non-beneficiary households. The regression analysis showed that wasting among children under-5 years in Malawi was affected by household participation in FISP, location of the household, the interaction of participation in FISP and Maize production, and the period in which the coupon was received. At a 5% level of statistical significance, there was a positive and significant correlation between households receiving a FISP coupon and the probability of having children under-5 years wasted.

**Table 5:** Results of impact of FISP on wasting among children under-5 years.

	**Wasting (logWHZ)** **b/se**
FISP	1.248** (0.659)
Maize1	0.061 (0.080)
legume1	−0.014 (0.018)
0.FISP#c.M~1	0.000 (.)
i.FISP#c.Maize1	−0.207** (0.112)
c.FISP_Seed#c.legume1	0.002 (0.003)
Age	0.007 (0.016)
Age2	0.000 (0.000)
reside	−0.105 (0.100)
edu_head	−0.006 (0.011)
1.r egion	0.000 (.)
2. region	0.204* (0.114)
3.region	0.188
	(0.118)
hhsize	0.033 (0.024)
2013.year	0.000 (.)
2016.year	0.550**** (0.112)
2019.year	−0.194** (0.088)
Sex	0.121 (0.105)
Year 2	−0.136 (0.203)
Year 3	−0.178 (0.166)
Sex_child	0.063 (0.060)
devage	0.001 (0.007)
devfarmsize	0.022 (0.037)
devsex	0.061 (0.165)
deveducation	−0.002 (0.019)
devhhsize	−0.039 (0.036)
Constant	−0.308 (0.535)
/	
sigma_u	0.363**** (0.067)
sigma_e	1.093**** (0.030)
Wald chi	107.688
Prob > chi2	0.000
Rho	0.100
Observations	1,662.000

WHZ was dummy for 1 = Yes and 0 otherwise. Results are significant at ****, **, * 0.1%, 1%, 5% and 10% levels respectively.

The impact of access to a FISP coupon on reducing wasting is only significant when we observe the maize output levels among beneficiary households. We find that households that accessed subsidized fertilizer and produced Maize had a lower probability of having children under-5 years wasted (*p* < 0.05).

The results further show an improvement in wasting levels of children under-5 years dwelling in households that received input coupons in the 2018/2019 season compared to the other seasons. We find that children under-5 years dwelling in households that received input coupons in 2015/2016 had a significantly higher probability of being wasted (*p* < 01). On the other hand, the probability of having children under-5 years wasted in households that received a coupon in 2018/2019 was significantly lower (*p* < 0.05)

In terms of region of residence, we find a positive and significant correlation of the probability of households having children under-5 years that are wasted if the household resides in the central region of the country (*p* < 0.10).

## Discussion

This study establishes evidence of how access to FISP affects household nutrition. The number of targeted beneficiaries in the samples used in this study dropped from 600 in 2013, 276 in 2016, and 145 in 2019 due to the government's reduction of the targeted beneficiaries and increased coupon redeeming fee (21). Overall, the mean wasting was 0.103 in 2013, −0.28 in 2016, and 0.173 in 2019. This finding shows that nutrition outcomes due to access to FISP coupons have been inconsistent, with better outcomes observed in 2013 and 2019.

Interestingly, our three-wave panel data finds that access to FISP coupons as a stand-alone predictor is not associated with reduced wasting values in households and does not directly translate into improved nutrition outcomes of wasting. Households that accessed FISP coupons had a higher probability of having children that were wasted. These results are different from what Karamba (56) and Harou (25) found, where the receipt of subsidized inputs led to a reduction in wasting (weight for height) among children under-5 years in beneficiary households. One possible explanation of these differences is the datasets used, i.e., Harou used two waves of 2012/13 and 2015/16 while Karamba used one wave of 2009/10. This suggests that while FISP aims to improve household food and nutrition security, the direct correlation might not always hold in all contexts. Another possible explanation for this phenomenon might be related to the selection criteria for the program, where households that receive FISP coupons are economically challenged, produce less food for consumption, and have less income to purchase food, which leads to children under-5 years being wasted.

Further on, a possible explanation could be related to the program's focus on Maize as a staple crop with less attention to other nutritionally high-value crops. Evidence shows that it is only in recent times that the program included legumes seed, but how far households are making use of this is yet to be verified. This finding could, however, be due to methodological specifications. We, therefore, tried to interact with the FISP (whether the household redeemed fertilizer and seed) variables with maize and legume production.

When we estimated the interacted effect of maize production conditioned on receiving and redeeming subsidized inputs, we observed a negative and significant correlation between wasting levels of children under-5 years (*p* < 0.01). These results, in principle, imply that nutritional benefits from FISP coupons are contingent on increased maize production, contributing to reduced wasting among children under-5 years old. This finding further implies that even if households have access to FISP coupons, the presence of other forms of constraints in accessing and utilizing additional resources required for higher maize production will likely result in undesirable nutrition outcomes. In light of the fact that no statistically significant impact was found for maize production on wasting, which concurs with Walls et al. (6), we suggest that the selection criteria for the FISIP beneficiaries should target economically challenged households with the appropriate supporting conditions to increase maize production.

We find no significant interaction effect for legumes and no evidence to support the pathway in our conceptual framework that legume production leads to dietary diversity and hence improved nutrition outcomes for children under-5 years in FISP households. Matita et al. (57) found a similar lack of evidence on the contribution of cultivating legumes to dietary diversity for households that accessed FISP coupons. However, a recent study finds a positive linkage between access and redemption of legume coupons with greater dietary diversity and evidence that the type of subsidized seed coupon matters for nutrition outcomes (58). Based on these two differing findings from the literature, we postulate that further analysis focusing on types of coupons redeemed and linkage with anthropometry outcomes should be considered in future studies.

We found that households that received a coupon in the 2015/2016 period had a significantly higher probability of having children under-5 years wasted. But households that received a coupon in the 2018/2019 period had a significantly lower likelihood of having children under-5 years wasted. These results can be understood in the context of the changes that have been implemented for the subsidy program (21, 57). Households that accessed coupons in 2015/2016 were the first group to pay higher redemption fees for the coupons, which could have affected the quantities of inputs redeemed by the vulnerable farming households.

We found that residing in the central region was associated with a household having a significantly higher probability of having children under-5 that are wasted. These results partly render to other findings that farmers in the central region have less diversified production systems, with Maize and tobacco continuing to dominate cultivation (58).

## Conclusions and recommendations

Our current study examines the link between FISP and its effect on the child malnutrition outcome and wasting of children under-5 years in Malawi. The FISP program continues to be a topic of interest due to the significant funding it receives within the Agricultural budget in the study country and the important role of Maize, the main targeted crop within the subsidy program. We find evidence that households that had received a coupon were overall associated with a higher probability of having children under-5 years with wasting. We further find those poor households that received input coupons (FISP) and produced Maize had better outcomes for wasting among children under-5 years. We also note that being a FISP beneficiary in the central region was associated with a higher probability of having children under-5 that are wasted. Across the sampling periods, we find that households receiving a coupon in 2015/2016 had a higher probability of having children under-5 years, while a lower probability was observed for the 2018/2019 period.

We understand that the use of Input Subsidy is not a stand-alone predictor that directly links to child malnutrition. Nevertheless, where beneficiary households can increase output of cereals such as Maize, important reductions in malnutrition outcomes such as wasting are attained. Based on the above results, we draw the following recommendations for further improvements in implementing the FISP in Malawi.

Firstly, our study highlights the important role that increased output plays in attaining nutritional outcomes such as wasting. We, therefore, propose that to achieve the desired impacts of input subsidy programs on nutrition outcomes, the targeting of beneficiaries needs to be based on the productivity levels of households. We acknowledge the intention of the government to support the highly resource-constrained households to improve food security and the economic wellbeing of the beneficiary households. Nevertheless, we suggest that for the government to achieve this noble objective, the targeted poor resource-endowed FISP beneficiary households should be supported with appropriate household level enabling conditions such as time-bound staggered social safety nets to ensure that they effectively put the inputs into use at the farm level for increased production of Maize and the other chosen crop enterprises. The revision of the targeting criteria for the FISP program initiated in the 2015/16 period and 2018/2019 period is an excellent step in the right direction toward this, and our results, where households that received FISP in 2019 had reduced wasting levels of children under-5, do indeed suggest that the revised criteria can generate significant positive outcomes from the FISP program.

Secondly, our results bring into question how the FISP program can best account for the spatial differences that exist across the three regions. The evidence from this study and other studies has shown that the nutrition outcomes linked to the FISP, such as dietary diversity and child anthropometry, are not consistent across the three regions in the country (58). While it is true that the targeted crops (Maize and legumes) are important crops at a country level, there is room for more conversations on whether the subsidy program should expand its targeted crops. Understanding the broader linkages that income pathways offer to access to important food crops can help streamline investments in other food systems within the regions that can be linked to the intended nutrition outcomes currently outlined in the FISP program.

## Potential study limitations and mitigation

As we argue in the paper, evaluating AIP subsidies needs to be a continuous effort to inform policy direction and implementation strategies. Our analysis only focused on the nutrition outcome of wasting. However, there is a need to assess how changes in the implementation of the FISP affect other nutrition outcomes both among children under-5 years and productive adults. Further, we acknowledge that further studies could identify more nuanced relationships by isolating the type of coupons redeemed by households.

## Data availability statement

Publicly available datasets were analyzed in this study. This data can be found here: https://microdata.worldbank.org/index.php/catalog/3819.

## Ethics statement

The studies involving human participants were reviewed and approved by Malawi National Statistic Office. The patients/participants provided their written informed consent to participate in this study. Written informed consent was obtained from the individual(s) for the publication of any potentially identifiable images or data included in this article.

## Author contributions

GT contributed to this study by suggesting the topic, data sourcing, data cleaning, file merging, and running the data analysis. EG was responsible for drafting the introduction sections, developing the conceptual framework, and drafting the discussion of results and final edits of the paper. BM improved the research objective and editorial works of the paper, including proper design of the tables, critiquing of the contents of the study, interpretation of the tabulated results, discussion of the research findings, and fine-tuning the recommendations. KM was responsible for reviewing the arguments and constructing linkages of the study findings with existing literature. SK developed the empirical methodologies used in the study. All authors contributed to the article and approved the submitted version.

## Funding

The project was funded by the African Economic Research Consortium (AERC) under the AFPON research project grant, Nairobi, Kenya, under the call Food security and Nutritional Outcome policy. The AFPRON research grant is one of the beneficiaries of Bill and Melinda Gates Foundation (BMGF) grant. The grant aimed at supporting research that investigates the link between national agricultural policies and nutrition pathways (INV-010015).

## Conflict of interest

The authors declare that the research was conducted in the absence of any commercial or financial relationships that could be construed as a potential conflict of interest.

## Publisher's note

All claims expressed in this article are solely those of the authors and do not necessarily represent those of their affiliated organizations, or those of the publisher, the editors and the reviewers. Any product that may be evaluated in this article, or claim that may be made by its manufacturer, is not guaranteed or endorsed by the publisher.
